# The Role of Elafin in the Pathogenesis of Psoriasis

**DOI:** 10.3390/ijms27041767

**Published:** 2026-02-12

**Authors:** Mateusz Matwiejuk, Agnieszka Kulczyńska-Przybik, Bartłomiej Łukaszuk, Hanna Myśliwiec, Piotr Myśliwiec, Adrian Chabowski, Barbara Mroczko, Iwona Flisiak

**Affiliations:** 1 Department of Dermatology and Venereology, Medical University of Bialystok, 15-540 Bialystok, Poland; 2 Department of Neurodegeneration Diagnostics, Medical University of Bialystok, 15-269 Bialystok, Poland; 3 Department of Physiology, Medical University of Bialystok, 15-222 Bialystok, Poland; 4 1st Clinical Department of General and Endocrine Surgery, Medical University of Bialystok, 15-276 Bialystok, Poland

**Keywords:** psoriasis, skin diseases, protein, elafin

## Abstract

Psoriasis is a chronic, immune-mediated inflammatory disease that affects the skin, nails, joints, and cardiovascular system. In this study, involving 50 psoriatic patients and 28 healthy controls (patients with inguinal hernia), serum elafin levels were measured using enzyme-linked immunosorbent assay (ELISA). The results revealed significantly higher serum elafin levels in the psoriatic group compared to healthy individuals. Moreover, we observed a statistically significant positive correlation between serum elafin levels and the Psoriasis Area and Severity Index (PASI) scores. These findings indicate that elafin—a protein involved in psoriasis pathogenesis—is significantly altered in the serum of psoriatic patients and may be associated with disease severity.

## 1. Introduction

Psoriasis is a chronic, non-contagious, immune-mediated disease that affects the skin, joints, and vascular system [1]. The prevalence of psoriasis varies significantly, from 0.51% to 11.43% in adults. In children, the estimated prevalence is lower, ranging from 0% to 1.37% [2]. Psoriasis can manifest at any age, with a notable proportion—between 25% and 40% of all cases—occurring during childhood [3]. There are two main known types of psoriasis: Type 1 and Type 2—classified based on factors such as age of onset, family history, and genetic background, particularly the presence of HLA-Cw6. Type 1 psoriasis (early-onset) typically develops before age 40, has a strong familial association, and is strongly linked to HLA-Cw6. It tends to be more severe than Type 2 (late-onset), which develops after age 40, lacks strong family history, and is not associated with HLA-Cw6 [4]. Genetic factors interact with environmental and intrinsic risk factors that modulate disease severity and lesion formation. Environmental triggers include mechanical stress (Koebner phenomenon), environmental pollution, medications (e.g., beta-blockers, lithium, antimalarials, non-steroidal anti-inflammatory drugs, angiotensin-converting enzyme inhibitors and abrupt withdrawal effects (for instance, from systemic corticosteroids), streptococcal and human immunodeficiency virus infections, and life style factors such as smoking and excessive alcohol consumption [5]. Intrinsic risk factors include metabolic syndrome, obesity, diabetes mellitus, dyslipidemia, hypertension, and psychological stress [6,7,8,9].

Based on clinical presentation, several types of psoriasis are recognized: plaque, guttate, inverse (flexural), and pustular psoriasis. Plaque psoriasis is the most common type and is characterized by erythematous or violaceous plaques with silvery-white scales. These lesions are often pruritic and may be painful or fissured [10]

Due to the systemic immune-mediated nature of psoriasis, it often coexists with multiple comorbidities, including psoriatic arthritis, cardiovascular disease, metabolic syndrome, inflammatory bowel disease, psychiatric disorders (such as depression, anxiety, and suicidal ideation), and malignancies. These include cutaneous T-cell lymphoma and non-melanoma skin cancers (NMSCs), such as squamous cell carcinoma partly due to the chronic use of phototherapy [11].

Interestingly, a study by Wu et al. [12] found that patients with psoriasis who also suffer from common comorbidities, such as mental health disorders, diabetes mellitus, hyperlipidemia, hypertension, liver disease, and obesity, tend to seek medical care more frequently than individuals without psoriasis. This indicates a greater burden on healthcare resources associated with this patient population. For instance, individuals with psoriasis and mental health disorders had an 8% higher rate of medical visits; diabetes—10% higher medical visit rate; hyperlipidemia—13% higher medical visit rate; hypertension—13% higher medical visit rate; liver disease—21% higher medical visit rate; and obesity—12% higher medical visit rate. All differences were statistically significant [12].

Moreover, data provided by Merola et al. [13] clearly illustrated the significant economic burden associated with psoriasis and psoriatic arthritis in comparison to individuals without these conditions. Based on a retrospective study from 2009 to 2020, conducted in the USA, annual all-cause healthcare costs per patient were $7470 for the control group, $11,062 for patients with psoriasis, and $29,742 for those with psoriatic arthritis [13].

The pathogenesis of psoriasis remains incompletely understood and is recognized as a complex, multifactorial process [14]. Current evidence supports the classification of psoriasis as a T-cell-mediated disease, with the IL-23/IL-17 axis playing a central and pivotal role [15,16]. Among the contributing factors, abnormalities in protein function appear to be of significant importance. Proteins are fundamentally involved in both the structural alterations of the skin and the dysregulated inflammatory responses characteristic of psoriasis [14].

A broad spectrum of protein classes contributes to the pathogenesis of psoriasis, beyond the T-cell-mediated immune response, including alarmins, antimicrobial peptides, autoantigens, cytokines, and growth factors. These proteins interact in complex and often overlapping pathways, driving inflammation, keratinocyte hyperproliferation, and structural skin changes [14]. T-cells also play a significant role in the inflammatory etiology of psoriasis; they are activated by upstream signals like IL-23. Subsequently, T-cells release key effector cytokines, including IL-17, IL-22, and interferon gamma (IFN-γ). These cytokines act directly on keratinocytes, inducing hyperproliferation and abnormal differentiation, particularly through the action of Il-22. Additionally, IL-17 promotes the release of pro-inflammatory mediators such as cytokines, chemokines, and host defense proteins, which may synergize with IFN-γ to further amplify the inflammatory cascade. The keratinocyte-derived mediators, in turn, recruit and activate additional immune cells—neutrophils, T-cells, and dendritic cells (DCs)—to the skin. This creates a self-perpetuating inflammatory loop, culminating in the chronic inflammation and epidermal thickening that typify psoriatic lesions [17].

Recent research has highlighted the emerging role of elafin in the pathogenesis and exacerbation of psoriasis [18,19,20,21,22]. Elafin is a human serine protease inhibitor that belongs to the chelonianin family of protease inhibitors [23]. In the 1990s, elafin (a 6-kDa protein) was first isolated from psoriatic skin. Its initial identification as a protein with elastase inhibitor features emphasized its potential relevance to this dermatosis [24,25]. Elafin is a potent inhibitor of neutrophil elastase, released by neutrophils during inflammation, contributing to tissue degradation. Moreover, it also inhibits the activity of pancreatic elastase and endogenous vascular elastase [26]. The upregulation of elafin in response to these pro-inflammatory cytokines (IL-1β and TNF-α) suggests a natural, protective feedback mechanism, responding by increased production of elafin [27,28]. Additionally, elafin exerts anti-inflammatory effects by suppressing nuclear factor-kappaB (NF-κB) activation in monocytes stimulated with lipopolysaccharide or lipoteichoic acid. This inhibition leads to decreased production of pro-inflammatory cytokines, reinforcing elafin’s potential regulatory role in immune responses [29].

Elafin contributes to the structural integrity of the cornified cell envelope of keratinocytes through the transglutaminase domain. Elafin’s incorporation into the cornified cell envelope helps reinforce the epidermal barrier, which is essential for preventing excessive water loss (transepidermal water loss—TEWL); blocking the entry of irritants, allergens, and microbes; and maintaining skin homeostasis. Overall, elafin plays a critical role in preserving the structural and functional integrity of the epidermis, which is notably compromised in psoriasis [18].

Interestingly, elafin also protects elastic fibers from enzymatic degradation. Its presence in abnormal and accumulated fibers is part of the pathological process of actinic elastosis, which is a feature of sun-damaged, aged skin. Elafin binds to elastin fibers to protect them from degradation caused by neutrophil elastase. In response to ultraviolet irradiation, dermal fibroblasts increase elafin expression, thereby enhancing resistance to elastolytic fiber breakdown, contributing skin aging [30].

Previous studies have demonstrated altered elafin expression in psoriatic lesional skin, suggesting its involvement in local disease mechanisms. However, psoriasis is increasingly recognized as a systemic inflammatory disorder rather than a disease confined to the skin. Despite extensive evidence regarding tissue-level elafin dysregulation, data on circulating elafin levels in patients with psoriasis remain limited. We therefore hypothesized that serum elafin levels may be altered in psoriasis and may reflect disease severity, providing insight into the systemic inflammatory component of the disease.

This article aims to deepen our understanding of elafin’s multifaceted role in the pathogenesis of psoriasis. To achieve this, we measured serum elafin concentrations in patients with psoriasis and compared them with levels in a healthy control group. We further examined the relationship between elafin levels and PASI scores, a standard measure of disease severity, as well as with various biochemical and clinical parameters.

## 2. Results

### 2.1. Study Population

This study included 50 patients (20 females and 30 males) with active plaque-type psoriasis and 28 healthy patients (24 females and four males). The control group consisted of individuals undergoing surgical treatment for inguinal hernia and without dermatological or systemic inflammatory diseases. The median age in the control group was 42 years (range: 37.5–47.2, normal distribution), while in the psoriatic group it was 51 years (range: 34.2–66.0, non-normal distribution). The mean duration of psoriasis was 16 years. In the control group, the median body mass was 69.5 kg (range: 63.8–79.2, normal distribution), the mean height was 165.0 cm (range: 161.5–170.2, non-normal distribution), and the median body mass index (BMI) was 25.1 kg/m^2^ (range: 23.5–27.9, normal distribution). Among the controls, 12 individuals (43%) had a normal weight, 11 (39%) were overweight, and five (18%) were classified as obese. In the psoriasis group, the median body weight was 84.0 kg (range: 75.5–95.8, normal distribution), the mean height was 172.5 cm (range: 164.2–176.0, normal distribution), and the median BMI was 29.0 kg/m^2^ (range: 23.9–31.8, normal distribution). Among patients with psoriasis 19 individuals (38%) were obese, 17 (34%) were overweight, and 14 (28%) had a normal weight. Based on the Psoriasis Area and Severity Index (PASI), disease severity in the psoriasis group was categorized as follows: seven patients (14%) had a mild form of psoriasis (PASI < 10), 26 patients (52%) had moderate psoriasis (PASI 10–20), and 17 patients (34%) had severe psoriasis (PASI > 20). Table 1 summarizes the main clinical features of the psoriatic group and the control group. In the following study, we relied on the same control and study groups as in the article published by Matwiejuk et al. [31].

### 2.2. Elafin Level

**Figure 1:**
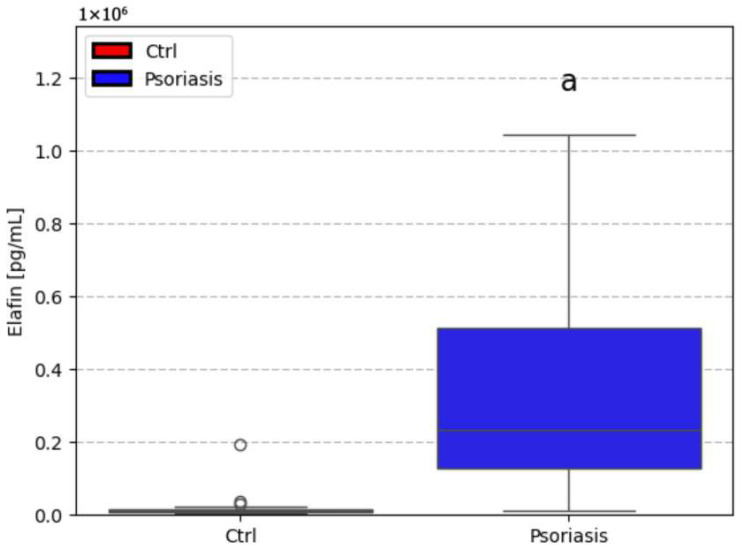
Comparison between elafin level in healthy (CTRL) patients’ serum and psoriatic patients’ serum [pg/mL]; a—difference vs. ctrl.

**Figure 2:**
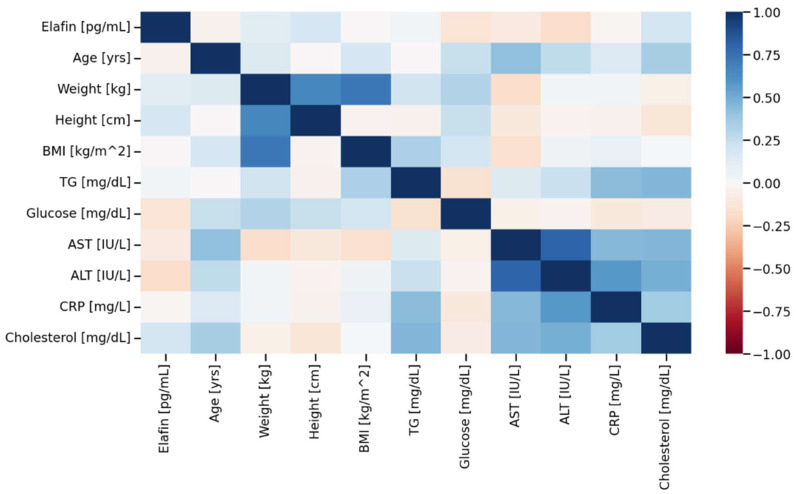
Correlation matrix (heatmap) in the control group. Pearson correlation coefficients are depicted as the shades of blue (positive correlation) or red (negative correlation). BMI—body mass index; CRP—C-reactive protein; TG—triacylglycerol; AST—aspartate transaminase; ALT—alanine transaminase.

**Figure 3:**
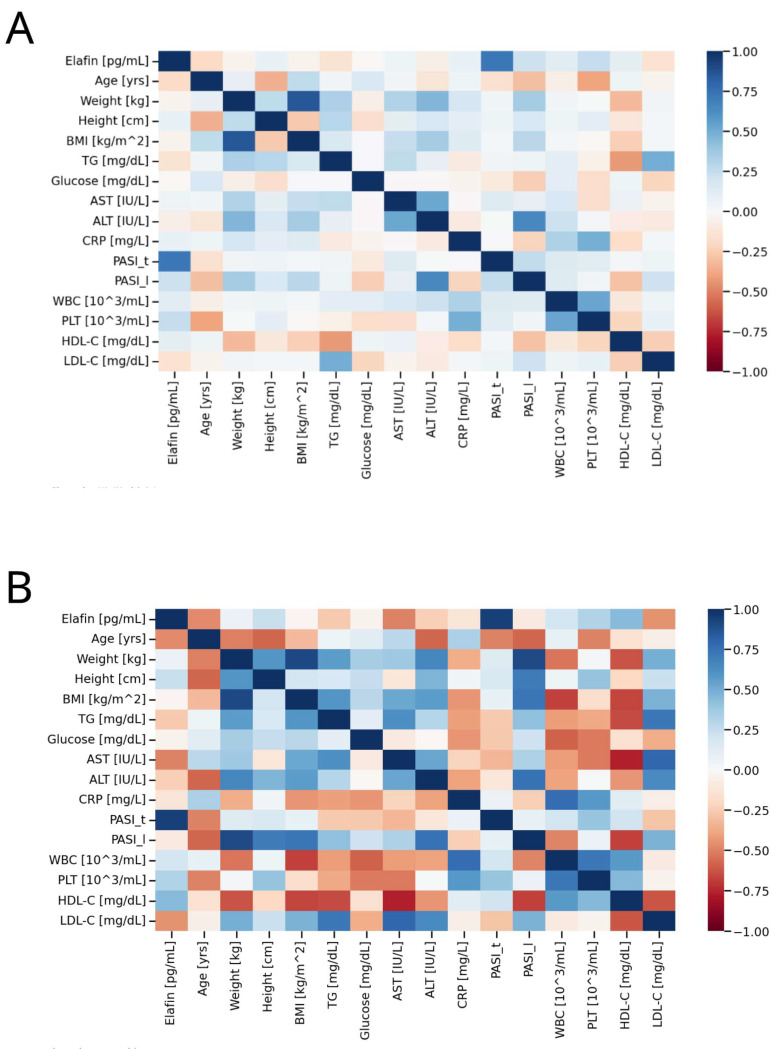
(**A**) Correlation matrix (heatmap) in the psoriatic group. (**B**) The heatmap shows a significantly positive correlation between elafin in the serum and psoriasis area and severity total (PASI) > 6 of patients with psoriasis. Pearson correlation coefficients are depicted as shades of blue (positive correlation) or red (negative correlation). BMI—body mass index; CRP—C-reactive protein; TG—triacylglycerol; AST—aspartate transaminase; ALT—alanine transaminase.

**Figure 4:**
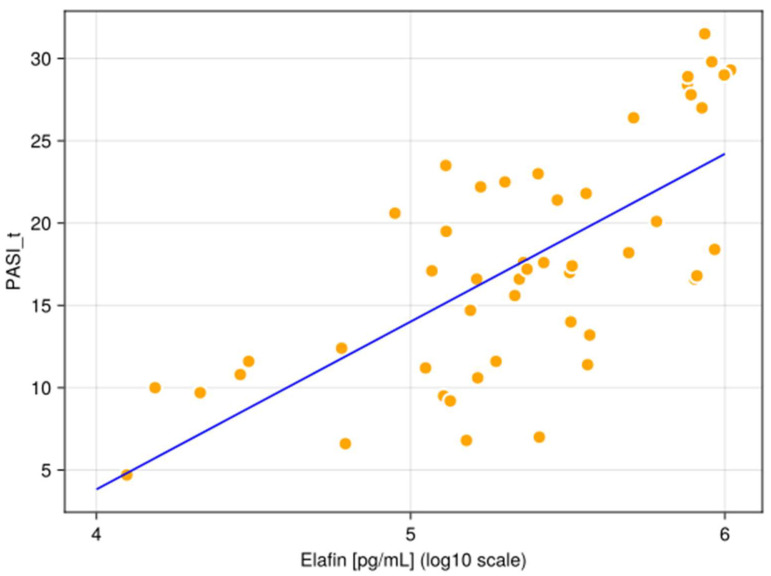
The scatterplot shows a correlation between elafin in the serum and Psoriasis Area and Severity Index (PASI) score of patients with psoriasis.

The median serum concentration of elafin found in psoriatic individuals (234,696.8 pg/mL, non-normal distribution) was significantly higher (*p* < 0.05) compared to healthy controls (12,072.0 pg/mL, non-normal distribution). Figure 1 illustrates the different in elafin levels between psoriatic patients and healthy individuals. Figure 2, Figure 3 and Figure 4 present various correlations observed in both the psoriatic and control groups, including relationships between elafin concentration and PASI scores, and selected clinical and biochemical parameters. In the control group, no significant correlations were found between elafin and other tested clinical parameters (Figure 2). In the psoriatic group, a significant relationship between elafin concentrations and PASI was noticed (Figure 3 and Figure 4).

## 3. Discussion

### 3.1. The Role of Elafin

In this study, we assessed serum elafin levels in patients with psoriasis and compared them with those of a healthy control group. In addition, we evaluated the potential link between elafin concentrations and various clinical and laboratory parameters within the psoriatic patient group, which, to the best of our knowledge, has not yet been comprehensively explored.

Our findings revealed that serum elafin levels were significantly elevated in patients with psoriasis in comparison to the serum samples of healthy individuals. Furthermore, we identified a statistically significant positive correlation between elafin and Psoriasis Area and Severity Index score in the psoriatic group (r = 0.94, *p* < 0.05), indicating a potential link between elafin and disease severity. These results are consistent with previous observations by Holmannova et al. [32] who also reported that patients with psoriasis have significantly higher serum levels of elafin compared to healthy individuals. The authors suggested that an increased elafin expression in psoriasis may contribute to both the inflammatory processes and the disruption of the epidermal barrier function. Importantly, the study proposed that elafin may serve as a useful biomarker, given its correlation with disease severity (PASI) and potential responsiveness to treatment. These findings support the utility of elafin not only in diagnosis and prognosis, but also in monitoring therapeutic outcomes in psoriasis [32].

Elgharib et al. [22] revealed in their study that significantly higher serum elafin levels were observed in psoriasis patients compared to healthy controls (2.47 ± 1.64 (0.6–6.2) vs. 0.61 ± 0.49 (0.01–1.5), *p* < 0.001, respectively), and notably, this elevation was observed even when considering smokers within the patient group and non-smokers in the control group. This implies that the increase in elafin is primarily linked to psoriasis itself, rather than solely to smoking status. Moreover, they spotted the correlation with CRP and erythrocyte sedimentation rate (ESR), which further solidifies the link between elafin levels and the systemic inflammatory burden in psoriasis. In addition, patients with a positive family history of psoriasis exhibited significantly higher elafin levels. This is an interesting finding, suggesting a potential genetic predisposition or a more aggressive inflammatory profile in those with a familial link to the disease, which is reflected in higher elafin [22].

In line with these observations, Albarazenji et al. [33] provided valuable evidence supporting elafin as a dynamic biomarker of treatment response. The study revealed that serum elafin levels significantly decreased following successful treatment with narrowband UVB (NB-UVB), suggesting that elafin levels reflect the disease’s activity and as a real-time indicator of therapeutic efficacy [33].

Similarly, Alghonemy et al. [34] reported that elafin has potential as both a biomarker for assessing disease activity and a therapeutic target for intervention in psoriasis. Patients suffering from psoriasis had significantly higher serum elafin levels (6.09 ± 8.91) compared with healthy controls 0.40 ± 0.35), with *p* less than 0.001. Moreover, the authors revealed that increased levels of inflammatory markers like ESR and CRP were observed in psoriasis patients, with *p* < 0.001, further supporting the inflammatory nature of the disease [34].

Elghetany et al. [35] further supported the previously reported findings on elevated elafin levels in psoriatic patients, confirming its correlation with disease severity and inflammation. Their study revealed that patients with psoriasis had higher level of elafin in serum in comparison to healthy controls (*p* < 0.001). Notably, they also examined the influence of factors like smoking status and positive family history, providing additional insights into the multifactorial interplay affecting elafin expression in psoriasis. These results further reinforce the concept of elafin as a promising biomarker in the pathophysiology of psoriasis [35].

Importantly, Alkemade et al. [19] emphasized that elafin in the serum is a more reliable and significant biomarker for psoriasis severity than urinary elafin. According to their findings, serum elafin levels demonstrated greater sensitivity and specificity in reflecting psoriasis activity, and they proposed that tracking elafin could be useful in monitoring response to treatment with cyclosporine A. Moreover, elafin’s responsiveness to treatment makes it a potentially predictive biomarker, and its involvement in disease mechanisms suggests it may also serve as a novel therapeutic target for future intervention strategies in psoriasis management [19].

Further histopathological and molecular evidence was provided by Nonomura et al. [20], who investigated elafin expression patterns at both the tissue and gene expression levels. Their analysis demonstrated marked upregulation of elafin in psoriatic lesions, particularly within the upper spinous and granular layers of epidermis. Maximal expression was marked near subcorneal microabscesses. Furthermore, even in the non-affected psoriatic epidermis, there was focal elafin expression in the subcorneal layer. These findings suggest that elafin is expressed in different psoriatic lesions. The authors strengthened the hypothesis that elafin is involved in multiple aspects of psoriasis pathogenesis: not only systemic inflammation, but also localized epidermal alterations, keratinocyte differentiation, skin barrier dysfunction, and the direct response to inflammatory cellular infiltrates [20].

Given the anti-inflammatory properties of elafin and its role as a serine protease inhibitor, therapeutic strategies in psoriasis would likely aim to enhance or restore its activity rather than inhibit it. By increasing elafin levels or function, it may be possible to counteract the inflammatory processes underlying psoriatic lesions, potentially complementing existing treatment approaches.

### 3.2. The Link Between Elafin and PASI

In our study, we identified a statistically significant positive correlation between serum elafin levels and PASI score above 6 (r = 0.41, *p* < 0.05). To the best of our knowledge, this specific threshold-based association has not been previously reported.

Consistent with our findings, Elgarib et al. [22], Albarazenji et al. [33], Alghonemy et al. [34], Elghetany et al. [35], and Alkemade et al. [19] all demonstrated a significant positive correlation between serum elafin levels and PASI scores in patients with psoriasis. These consistent findings collectively support the hypothesis that elevated elafin concentrations are associated with increased severity of psoriatic skin lesions, as measured by the PASI.

Interestingly, although elafin is traditionally recognized for its anti-inflammatory properties [36], its positive association with PASI scores, particularly in moderate-to-severe disease (PASI > 6), may seem paradoxical at first glance. However, this phenomenon may reflect a compensatory upregulation of elafin in response to heightened inflammatory activity in psoriasis, which is a chronic immune-mediated disorder involving not only the skin, but also joints and the vasculature [37].

The PASI remains the most widely accepted tool for quantifying disease severity in both clinical and research settings. It does not just measure the extent of the lesions, but also grades their severity based on key characteristics. The PASI takes into account the percentage of the body covered by psoriasis. Moreover, it involves the severity of lesions, which consists of grading three key clinical signs on a scale (usually 0–4 for none to very severe) within each affected area: erythema, induration, and desquamation. The final PASI score is a calculated value, ranging from 0 (no disease) to 72 (maximal disease). Generally, a PASI score of 5 to 10 is considered moderate disease, and a score over 10 is considered severe [38].

Elafin appears to offer a notable advantage in assessing the severity of skin lesions in the course of psoriasis in comparison to the PASI score alone. Using serum elafin levels alongside the PASI score can address several limitations of the PASI in assessing psoriasis severity. The PASI, while widely used, has some noteworthy drawbacks. First, PASI scores rely on visual assessments of erythema, scaling, and plaque thickness, which can be affected by environmental features like temperature, humidity, or topical treatments, leading to inconsistent evaluations. Second, the PASI does not prioritize high-impact areas such as the face, hands, or feet, which can significantly affect quality of life but may contribute minimally to the overall score. Third, PASI scores above 35 are rare, causing half of the PASI scale to be underutilized and less discriminative for severe cases. Fourth, the PASI focuses on cutaneous manifestations and does not cover the broader inflammatory state of the patient, which is critical in systemic diseases like psoriasis [22].

Nicolescu et al. [39] reported that the PASI and BSA may underestimate disease burden in anatomically complex or difficult-to-treat areas, such as the scalp, nails, palms/soles, and genital region. Notably, serum elafin may correlate differently with specialized severity indices (e.g., PSSI or NAPSI) or with quality-of-life measures in these difficult-to-treat sites, potentially providing additional biological insight where conventional clinical scores are limited. Furthermore, the authors emphasized the importance of nuanced severity assessment in guiding therapeutic decision-making. This perspective could be incorporated into the discussion by proposing that biomarkers such as elafin may help refine disease severity assessment beyond traditional indices [39].

Standard indices such as the PASI and BSA are widely used to assess psoriasis severity; however, they might not fully capture the true disease burden in hard-to-treat areas like the scalp, nails, palms/soles, and genital region [39]. While we observed a positive correlation between serum elafin and the PASI, it remains possible that circulating elafin could correlate differently with specialized regional indices, such as the PSSI or NAPSI, or with patient-reported quality-of-life measures in anatomically complex sites. Future investigations should explore whether serum elafin levels reflect both conventional and specialized severity scores. Such studies could clarify whether biomarkers like elafin may complement clinical indices and provide additional insight into disease burden, particularly in patients with psoriasis concentrated in difficult-to-treat areas.

Our findings extend previous tissue-based studies by demonstrating that elafin alterations in psoriasis are not restricted to the local cutaneous environment but are also detectable at the systemic level. The observed correlation between serum elafin levels and PASI scores suggests a potential association between circulating elafin and overall disease activity.

While these results do not establish causality or disease specificity, they support the concept that elafin may represent a link between local epidermal inflammation and systemic immune activation in psoriasis. Further studies are required to elucidate the mechanistic role of circulating elafin and to determine its potential utility as a biomarker of disease severity.

However, several limitations of our research should be mentioned.

First, the study cohort was relatively small (50 psoriatic patients and 28 controls), which curbs the statistical power and generalizability of the findings. Nonetheless, the significant differences observed between groups and the robust correlation with disease severity suggest that the findings are meaningful. Future studies with larger, multicenter cohorts are warranted to confirm these results and to provide more precise effect size estimates. Second, all blood samples were collected before the initiation of psoriasis-specific treatment, and it is unclear how systemic therapy might subsequently affect the elafin level in the serum. Third, we found out that there is a positive, significant correlation between elafin in the serum of psoriatic patients and PASI > 6, but we did not observe any correlation for PASI < 5. In this case, we could not predict the potential correlation between serum level of elafin and a mild course of psoriasis. Fourth, the lack of comparison of serum elafin levels with those in other dermatoses makes it difficult to demonstrate strict specificity for psoriatic pathogenesis. Larger, longitudinal studies are needed to prove these outcomes and to more clearly clarify the underlying mechanisms.

A limitation of the present study is the inclusion of healthy controls only, without comparison to other inflammatory skin diseases. Consequently, it cannot be determined whether the observed elevation of serum elafin is specific to the pathogenesis of psoriasis or reflects a non-specific marker of cutaneous inflammation. Therefore, while our findings demonstrate an association between increased serum elafin levels and psoriasis, they should not be interpreted as evidence of disease specificity. Elevated elafin may represent a broader inflammatory response rather than a psoriasis-exclusive mechanism. Future studies directly comparing patients with psoriasis to those with other inflammatory dermatoses, such as atopic dermatitis or hidradenitis suppurativa, are warranted to clarify the disease specificity and potential biomarker utility of serum elafin in psoriasis.

An additional limitation of this study is the difference in body mass index between patients with psoriasis and controls. Obesity is a common comorbidity in psoriasis and is itself associated with systemic low-grade inflammation. Therefore, the potential influence of BMI on circulating elafin levels cannot be fully excluded.

Future studies with BMI-matched control groups or multivariable analyses adjusting for metabolic factors are needed to further clarify the independent relationship between serum elafin and psoriasis severity.

## 4. Materials and Methods

A total of 50 patients (20 females and 30 males) with active plaque-type psoriasis, at a median age of 51.0, and 28 healthy controls (24 females and 4 males) at a median age of 42.0 were enrolled in the study. The severity of psoriasis was estimated using the PASI [40]. BMI was calculated based on self-reported weight and height [41].

Demographic and clinical characteristics, including age, sex, and BMI, were recorded for all participants. While age and sex distributions were comparable between the psoriasis and control groups, BMI was higher in patients with psoriasis, consistent with the known epidemiological association between psoriasis and obesity.

None of participants followed dietary restriction. Patients with a history of chronic conditions such as hypertension, liver diseases (including non-alcoholic fatty liver disease), cardiovascular disease, or diabetes mellitus, as well as those with abnormal laboratory test results were excluded based on hospital records. Laboratory tests were conducted prior to any treatment initiation. All participants provided written informed consent before enrolment. The study protocol was approved by the local university bioethical committee (no APK.002.272.2025) and followed the principles of the Helsinki Declaration. Peripheral blood samples were collected following an overnight fast and prior to treatment. After centrifugation, serum samples were stored at −80 °C until further analysis.

### 4.1. Elafin Analysis

The concentration of elafin (pg/mL) in serum was assessed by the commercially available Human Trappin-2 (PI3) Elisa kit Invitrogen (Thermo Fisher Scientific, Frederick, MA, USA) (cat. nr. EHPI3, analytical sensitivity 5 pg/mL). Quantitative determination of elafin was performed according to the manufacturer’s instructions in the Department of Neurodegeneration Diagnostics. Serum samples were diluted 1:200. Standards and samples were measured in duplicate, ensuring a coefficient of variation (CV) below 20%. Absorbance was read at 450 nm using a microplate reader.

### 4.2. Statistical Analysis

The sample size estimation was performed at the onset of the investigation. Thus, type I error was set at α ≤ 0.05 and power was set to be ≥0.8. The data in Table 1 are presented as the median and interquartile range (first and third quartile). Categorical variables (Table 1) were presented as counts and were compared using χ2 test with Yates continuity correction. The data presented on boxplots are expressed as median (middle horizontal bar), interquartile range (box), and whiskers (1.5 * IQR). The between group comparisons for continuous variables (boxplots and Table 1) were made with Student’s t-test or Wilcoxon test. The choice of the test was made based on the fulfillment of normality (assessed with Shapiro–Wilk’s test) and variance homogeneity (estimated with Levene’s test) criteria. The correlation analysis (heatmaps) were constructed based on the Pearson’s correlation coefficients. The obtained *p*-values for correlations were adjusted for multiple comparisons (Benjamini–Hochberg correction). A set of selected statistically significant correlations was presented on scatterplots with a trend line (obtained from linear regression) overlaid on them. The obtained *p*-value < 0.05 was deemed to be statistically significant.

## 5. Conclusions

Our results indicate that elevated elafin levels in patients with psoriasis, compared to healthy controls, suggest a broader role for this protein in this protein in the systemic pathogenesis of psoriasis. This finding supports the notion that inflammatory abnormalities in the course of psoriasis extend beyond visible skin lesions, implicating circulating proteins such as elfin in the development of this dermatosis. These insights s highlight elafin’s potential as a biomarker and possibly investigate elafin as a potential future therapeutic target to inhibit the inflammatory processes underlying psoriasis. However, given the complex pathogenesis of psoriasis and the limitations of our study, further large-scale research is needed to clarify elafin’s precise functions and therapeutic potential in this context.

Moreover, we observed a statistically significant positive correlation between serum elafin levels and PASI scores in patients with psoriasis. While elafin is traditionally considered an anti-inflammatory protein, its functional role in chronic inflammatory conditions like psoriasis may be altered or subverted. Considering that elafin inhibits elastase activity, thereby protecting the skin from extracellular matrix degradation and exacerbation of inflammatory processes, its elevated expression in psoriasis is likely a compensatory mechanism activated by the body in response to increased protease activity and chronic inflammation. This unexpected correlation emphasizes the critical need for more research into the functional state of anti-inflammatory mediators in chronic inflammation. Larger, prospective studies are essential to determine whether elafin could serve prognostically or therapeutically in the management of psoriasis.

## Figures and Tables

**Table 1:** Clinical and biochemical characteristics of the control group (CTRL) and psoriatic patients (PSO). Data are presented as median and interquartile range. a—statisticaly significant difference vs. CTRL (*p* < 0.05); BMI—body mass index; CRP—C-reactive protein; TG—triacylglycerol; AST—aspartate transaminase; ALT—alanine transaminase.

Clinical and Laboratory Features	CTRL	PSO
**Age [years]**	42.0 (37.5–47.2)	51.0 (34.2–66.0)
**Weight [kg]**	69.50 (63.8–79.2)	84.0 (75.5–95.8) a
**Height [cm]**	165.0 (161.5–170.2)	172.5 (164.2–176.0) a
**BMI [kg/m^2^]**	25.1 (23.5–27.9)	29.0 (23.9–31.8) a
**CRP [mg/dL]**	1.0 (1.0–2.0)	3.2 (1.5–6.9) a
**Glucose [mg/dL]**	86.5 (78.8–91.0)	85.0 (80.0–93.0)
**TG [mg/dL]**	73.0 (67.5–82.0)	116.0 (86.2–134.5)
**AST [U/L]**	17.5 (15.0–21.0)	20.0 (16.2–27.0) a
**ALT [U/L]**	15.5 (11.5–18.2)	19.0 (14.2–27.8) a
**Sex [no. female/no. male]**	24/4	20/30 a

## Data Availability

Data available upon request from the corresponding author.

## Abbreviations

The following abbreviations are used in this manuscript:

DCs	Dendritic cells
ELISA	Enzyme-linked immunosorbent assay
PASI	Psoriasis Area and Severity Index
NMSC	Non-melanoma skin cancer
IFN-γ	Interferon gamma
TEWL	Transepidermal water loss
BMI	Body mass index
ESR	Erythrocyte sedimentation rate
